# Molecular Immunotherapy: Promising Approach to Treat Metastatic Colorectal Cancer by Targeting Resistant Cancer Cells or Cancer Stem Cells

**DOI:** 10.3389/fonc.2020.569017

**Published:** 2020-11-09

**Authors:** Stefan Forster, Ramin Radpour

**Affiliations:** ^1^ Tumor Immunology, Department for BioMedical Research (DBMR), University of Bern, Bern, Switzerland; ^2^ Department of Medical Oncology, Inselspital, Bern University Hospital, University of Bern, Bern, Switzerland

**Keywords:** colorectal cancer, cancer stem cells, metastasis, cancer therapy, immunotherapy, immune-checkpoint inhibitors

## Abstract

The immune system is able to recognize and eliminate tumor cells. Some tumors, including colorectal cancer (CRC), induce immune tolerance *via* different mechanisms of “immunoediting” and “immune evasion” and can thus escape immune surveillance. The impact of immunotherapy on cancer has been investigated for many years, but so far, the application was limited to few cancer types. Immuno-oncological therapeutic strategies against metastatic colorectal cancer (mCRC), the adaptive immune system activating approaches, offer a high potential for adaptation to the great heterogeneity of CRC. Moreover, novel treatment approaches are currently being tested that might specifically target the disease initiating and maintaining population of colorectal cancer stem cells (CSCs). In this review, we aim to summarize the current state of immune-oncology and tumor immunotherapy of patients with mCRC and discuss different therapeutic modalities that focus on the activation of tumor-specific T-cells and their perspectives such as tumor vaccination, checkpoint inhibition, and adoptive T-cell transfer or on the eradication of colorectal CSCs.

## Introduction

Colorectal cancer (CRC) is the third most common cancer and the fourth most common cause of cancer-related deaths (1). Worldwide, 1.4 million people fall ill every year and almost 700,000 people die due to metastatic CRC (mCRC) (2, 3). The majority of CRC patients develop metastases during the course of the disease, which is associated with a dismal prognosis and a 5-year survival rate of less than 10% (4). Approximately 15–25% of patients present with liver metastases at the time of initial diagnosis and 30% develop liver metastases later in the disease period (5). Despite different systemic therapy advances, more than 80% of patients with mCRC die within 5 years upon diagnosis. Currently, the majority of mCRC patients are treated with a combination of a biological agent together with a cytotoxic drug. While chemotherapy combined with surgical rehabilitation and/or radiological interventional procedures are the treatment of choice, several clinical parameters (e.g., age, comorbid illness, tumor localization, tumor burden, and resectability) influence the treatment options. Among those clinicopathological parameters, molecular characteristics of CRC including B-raf proto-oncogene, serine/threonine kinase (*BRAF*), human epidermal growth factor receptor 2 (*HER2*), microsatellite instability (MSI), and rat sarcoma homolog family (RAS) are important therapy determinants (5, 6). Resection and (neo) adjuvant chemotherapy (CTx) can improve 5-year survival rates (7). However, the recurrence rate is 40–75%, of which 50% affect the liver (8). With extensive liver metastases, surgical procedures using established technologies are often no longer possible, and the option of surgical rehabilitation is reserved for only a minority of these patients (9). The presence of non-resectable colorectal metastases implies a significantly worse prognosis. Under this condition, the palliative CTx remains the only therapeutic option left (10). In order to prevent tumor progression, immunotherapy approaches are proposed. In principle, the procedure should be determined for all patients with mCRC in interdisciplinary tumor boards.

Cancer stem cells (CSCs) represent a minor fraction of the bulk tumor cell population that could potentially reconstitute and propagate the disease. CSCs are found in different tumor types including colorectal tumors (11–14). CSCs can also induce tumors in foreign tissues (xenograft models); they diverge in different tumor types by their specific cell surface markers and have the potential to rebuild heterogeneous tumor tissue. In addition, CSCs possess stem cell properties such as self-renewal and quiescence that are regulated by cell-intrinsic and cell-extrinsic mechanisms (15). CSCs are mainly resistant to conventional therapies such as chemotherapy, irradiation and against immune attack; therefore, they are the main initiator of cancer relapse after primary treatment. This may be due to the different escape mechanisms of CSCs and/or due to the protective mechanisms of the microenvironment, e.g., the tumor niche (14, 16). For this reason, targeting and eradication of CSCs have been some of the main challenges in cancer treatment (17).

The immunotherapy of tumors has established itself as an important pillar of oncological treatment. Distinction of immune-based therapies is made according to their mechanism of action between active and passive immunotherapies. Decades of preclinical and clinical research suggest that the immune system is able to prevent tumorigenesis and fight cancer (18). The immunosuppressive function of inhibitory cytokines/chemokines as well as the complex microenvironment of CRC, reduce the immune function to promote CRC growth (6).

In CRC, an accumulation of driver mutations leads to the formation of the tumor-initiating cells, that represent a “foreign tissue” to the immune system. To date, the immunotherapy benefit is mainly confined to a small subset of patients with hyper mutated microsatellite instability-high (MSI-H) tumors such as CRC patients with deficient mismatch repair (dMMR) who only represent a small proportion of CRC/mCRC patients. Those dMMR tumors carry a high level of somatic mutations and therefore are considered as being highly immunogenic (6).

## Active and Passive Immunotherapies

**Figure 1:**
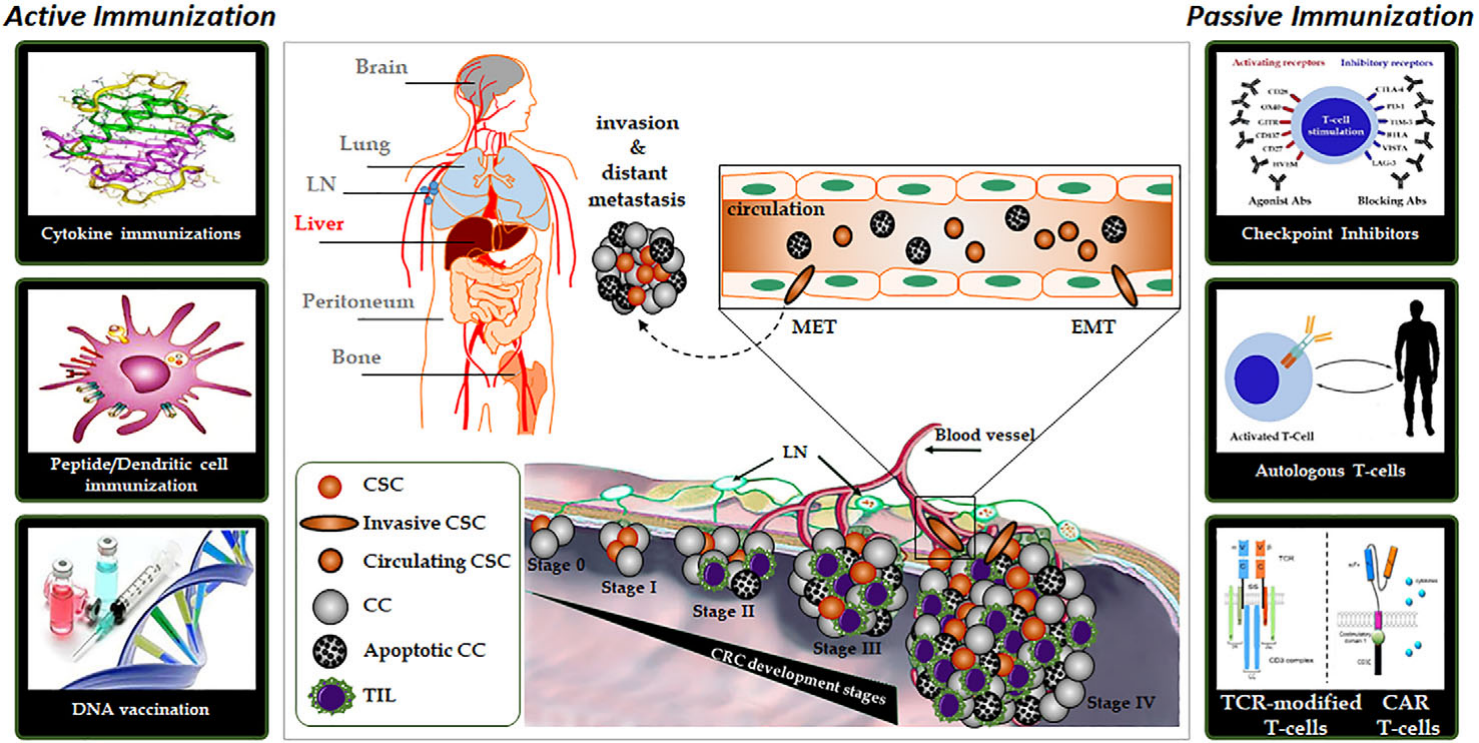
The complex organization of cancer initiation, progress, and distant metastasis for colorectal cancer and important active or passive immunotherapy approaches. Abbreviations: CC, Cancer cell; CSC, Cancer stem cell; EMT, Epithelial-mesenchymal transition; MET, Mesenchymal-epithelial transition; LN, Lymph nodes; TIL, tumor-infiltrating lymphocytes.

In general, immunotherapies can be divided into active and passive forms. The difference is whether the molecule used for treatment works by activating the immune system or is only part of the immune system itself (**Figure 1**).

## Active Immunotherapies

### Cytokines

The discovery, cloning and recombinant production of intercellular messengers, known as cytokines, was initially marked by the hope that they could be used to treat tumors (19). However, it became clear that sometimes their high potency could cause systemic side effects before inducing a tumor-directed effect. For example, interleukin-1β (IL1B) already leads to fever attacks at the nanogram level (20). Only three representatives of this category are currently clinically used in oncology: interleukin-2 (IL2), interferon-α (IFNA1), and tumor necrosis factor-α (TNF). IL2 is predominantly involved in the activation of various lymphocyte populations [NK (natural killer) and T-cells]; it induces and reactivates antitumoral immune responses. Although this type of therapy shows numerous side-effects, treatment with IL2 resulted in persistent remissions in a subset of patients with mCRC (21). Moreover, IL2 is frequently used in immunotherapy combinations involving autologous T-cells to induce T-cell expansion (22). IFNA1 is approved under defined conditions for the treatment of a variety of tumor types including mCRC alone or in combinations (23). The recombinant IFNA1 mimics a viral infection and leads to an antiviral program in the patient and its tumor tissue. In this case, IFNA1 presumably acts directly on the tumor cell and on its environment and thereby unfolds its therapeutic effect (24). The therapeutic window is narrow due to the high toxicity of the substance. TNF triggers both an antitumor inflammation and a direct cytotoxic effect. However, it has been reported that Th17-type cytokines (including TNF and IL6) promote CRC growth *via* activation of *NFKB1* and *STAT3* genes (25).

### Tumor Vaccination

Vaccination leads to the detection of tumor antigens by the immune system, subsequently triggering a specific antitumor immune response. In tumor vaccination, the presentation of tumor antigens allows effective activation of tumor-specific T-cells (i.e., CD8^+^ cytotoxic T-cells), thereby inducing or increasing an antitumor immune response.

### Agonists for Pattern Recognition Receptors

Pattern recognition receptors are important components of the innate immune response. They are used for the rapid detection of bacteria and viruses *via* the binding to specific patterns of these pathogens. This triggers pro-inflammatory signaling cascades that first mobilize soluble and cellular components of the innate immune response. The activation of pattern recognition receptors may also lead to the induction of an adaptive, acquired immune response. With the discovery of these receptors and their ligands, it was suggested that such agonists could be used for tumor therapy. As an example, catumaxomab binds on the one hand to the T-cell antigen CD3 and on the other hand to EPCAM (“epithelial cell adhesion molecule”), a tumor-associated antigen (26). Via its CD3 binding arm, catumaxomab activates T-cells by cross-linking them with tumor cells thus leading to tumor cell lysis. In addition, catumaxomab has also a functional Fc domain. Via this Fc domain, catumaxomab binds to antigen-presenting cells, possibly promoting the development of an immunological memory. The second approved product is blinatumomab, a bispecific antibody that binds to CD3 and CD19. This has the peculiarity that it consists of two so-called “single chain domains” (27). Catumaxomab and blinatumomab are examples of how T-cells can be targeted against tumors.

### Target Antigens for Tumor Vaccination

In tumor vaccination, highly complex, polyvalent and inaccurately characterized antigenic mixtures or well-defined antigens (Ag) can be used alone or in combination as vaccines. Frequently used Ags in clinical studies are Ag overexpressed in tumor cells, so-called tumor-associated antigens (TAA), cancer-testis Ag and oncofetal Ag (**Table 1**). Although tumor-individual and patient-specific Ags, so-called neoantigens, have been known for a long time, they can only be exploited by high-throughput screening/sequencing methods including the help of dedicated software and bioinformatic algorithms to predict the peptide binding avidity to MHC molecules (28). Vaccination strategies against patient-specific neoantigens appear promising today. The concept of neoantigen vaccines is currently being investigated in different clinical studies for CRC (**Table 2**).

**Table 1:** Potential tumor antigens for CRC vaccination.

Antigen	Examples	Strength	Weakness
Tumor-associated antigens (TAA)	PSG2 (CEA), ERBB2 (Her2/neu), GP100, MLANA (MART-1), MUC1, PSA, Tyrosinase	Immunogenic with strong expression in many tumors and low expression in normal tissue	It is possible that only T-cells with weak avidity are activated
Mutated tumor-specific antigens (neoantigens)	TP53, RAS, patient-specific mutations	Activation of T-cells with high avidity and effectiveness	Identification so far very cost-intensive, therefore not yet applicable for the routine application
Cancer-testis antigens	MAGE (e.g., MAGEA3), CTAG1B	Immunogenic with expression in numerous tumors	It is possible that only T-cells with weak avidity are activated
Oncofetal antigens	AFP	Immunogenic with strong expression in many tumors, no expression in adult normal tissue	It is possible that only T-cells with weak avidity are activated

AFP, alpha fetoprotein; CEA, carcinoembryonic antigen; CTAG1B, cancer/testis antigen 1B; ERBB2, erb-b2 receptor tyrosine kinase 2; Her2/neu, human epidermal growth factor receptor 2; MART-1, melanoma antigen recognized by T-cells; GP100, glycoprotein 100; MAGE, melanoma antigen-encoding gene; MUC1, mucin 1; PSA, prostate-specific antigen; PSG2, pregnancy-specific beta-1-glycoprotein 2; TP53, tumor protein P53.

**Table 2:** Overview of immunotherapy approaches and clinical trials in CRC/mCRC.

Group	Target type	Target molecule	Treatment strategy	Therapy form	Combination partners	No. of patients	Study group	Phase	Ref.
**Protein/Peptide Immunization**	TAA/TSA	CEA and CRC neoantigens	Peptide-loaded DC (DC vaccination)	M	–	25	MSI-high CRC and Lynch Syndrome	I/II	[NCT01885702]
	TSA	CRC neoantigens	Listeria monocytogenes based vaccine	M and C	Pembrolizumab	48	mCRC (Stage IV)	I	[NCT03265080]
	TSA	CRC neoantigens	Peptide-loaded DC (DC vaccination)	C	IL2	19	mCRC (Stage IV)	II	[NCT02919644]
	TAA/TSA	CRC neoantigens	Synthetic tumor-associated peptide vaccine	C	Imiquimod,Pembrolizumab	60	mCRC (Stage IV)	I	[NCT02600949]
**Autologous T-Cell Therapy**			PDCD1-activated autologous T-lymphocytes (PDCD1-T)	C	Bevacizumab, XELOX	284	mCRC (Stage IV)	III	[NCT03950154]
			Autologous Neo TCR-T Cells (Neo TCR-P1)	C	Nivolumab	148	mCRC (Stage IV)	I	[NCT03970382]
			Autologous tumor-infiltrating lymphocytes (MDA-TIL)	C	IL2	60	Recurrent or refractory CRC	II	[NCT03610490]
**CAR-T cells**		EGFR	EGFR-targeted-CAR-T cells	M	–	20	EGFR-positive mCRC (Stage IV)	I/II	[NCT03152435]
		PSG2 (CEA)	Anti-CEA-CAR-T cells	M	–	75	Relapsed/refractory CEA+ CRC	I	[NCT02349724]
		EPCAM	Anti-EpCAM-CAR-T cells	M	–	60	Relapsed/refractory EpCAM+ CRC	I/II	[NCT03013712]
		KLRK1 (NKG2D) ligands	NKG2D-based CYAD-CAR-T cells	C	FOLFOX	36	mCRC (Stage IV)	I	[NCT03692429]
		MET	Anti-MET CAR-T cells	M	–	73	c-MET positive CRC	I/II	[NCT03638206]
**Immune-Checkpoint Inhibitors**		PDCD1 and CTLA4	Anti-PDCD1 (Nivolumab) Anti-CTLA4 (Ipilimumab)	C	Temozolomide,Nivolumab,Ipilimumab	100	mCRC (stage IV)microsatellite stable	II	[NCT03832621]
		PDCD1	Anti-CD247 (Durvalumab)	C	Pexidartinib	48	Advanced or mCRC (Stage IV)	I	[NCT02777710]
		CD247	Anti-CD247 (Avelumab)	C	Cetuximab,Irinotecan	59	mCRC (Stage IV)	II	[NCT03608046]
		CD247	Anti-CD247 (Avelumab)	M	–	402	CRC (Stage III) dMMR	III	[NCT03827044]
		PDCD1	Anti-PDCD1 (Pembrolizumab)	M and C	INCB001158	424	mCRC (stage IV)	I/II	[NCT02903914]
**Cytokines**			L19TNFa	M	–	34	mCRC (stage IV)	I/II (compl.)	[NCT01253837]
			Recombinant IFAB	C	Celecoxib, Rintatolimod	12	mCRC (stage IV) or recurrent CRC	II	[NCT03403634]
			Interleukin-2	M	–	27	mCRC (stage IV)	II (term.)	[NCT00176761]
**DNA/RNA Vaccination**		CEA	CEA-RNA pulsed DC cancer vaccine	M	–	22	mCRC (stage IV)	I/II (compl.)	[NCT00003433]

C, combination therapy; CAR-T cells, chimeric antigen receptor T cells; CD247 (PDL-1), programmed cell death-ligand1; Compl., completed; CRC, colorectal cancer; DC, dendritic cells; dMMR, deficient mismatch repair; IFAB, interferon Alfa-2b; IL2, interleukin-2; KLRK1, killer cell lectin-like receptor K1; M, monotherapy; mCRC, metastatic colorectal cancer; MSI, microsatellite instability; PDCD1, programmed cell death-rpotein-1; PSG2, pregnancy-specific beta-1-glycoprotein 2; TAA/TSA, tumor-associated antigens/tumor-specific antigens; TIL, tumor-infiltrating lymphocytes; term, terminated.

### Tumor Vaccination Strategies

Tumor vaccination involves a wide range of approaches, which can be essentially divided into three strategies: peptide/protein vaccines, cell-based vaccines, and genetic vaccines. In peptide/protein vaccination, peptides or proteins from tumor antigens are administered as vaccines in combination with different adjuvants (29). Previous peptide/protein vaccination studies have often used MHC class I-restricted peptide epitopes from TAA. However, most peptide/protein-based vaccine research approaches have yielded disappointing results so far and have not been further developed until their clinical testing (30).

In cell-based vaccination, cells or cell lysates serve as a vaccine. Here, vaccination strategies are mainly based on autologous dendritic cells (DC), the most effective antigen presenting cells (APC). In this strategy, DC progenitor cells are taken from the blood of the patient, are cultivated *in vitro* and stimulated by the addition of tumor-specific antigens. These pre-treated cells are then reinfused into the patient (30). Several DC/APC-based vaccination strategies are in advanced clinical trials. Other cell-based vaccine approaches, such as vaccination with autologous or allogeneic *ex vivo* irradiated tumor cells, have shown disappointing results in previous studies (30).

**Figure 2:**
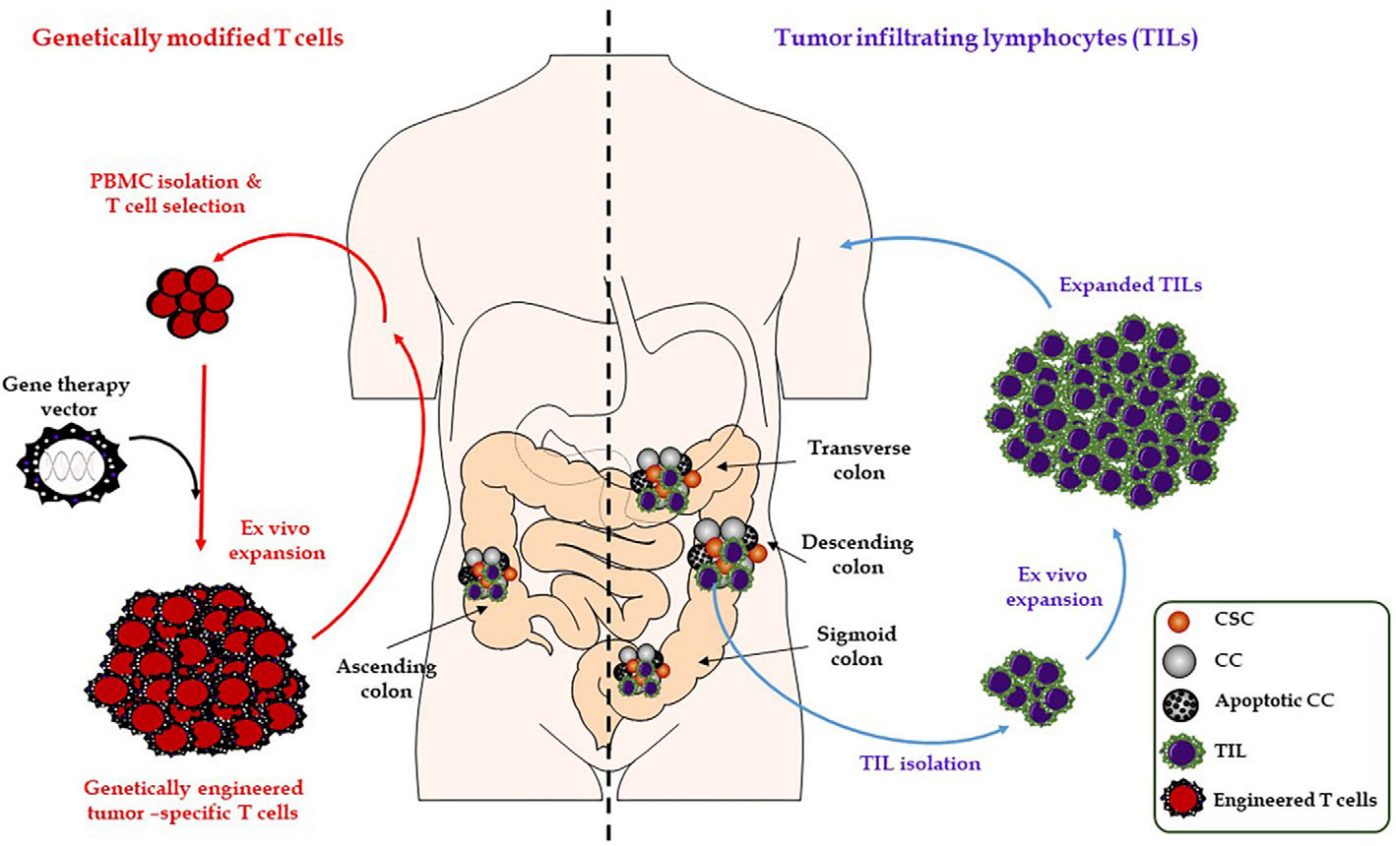
Illustration of adoptive T-cell transfer. Adoptive transfer of TIL (right). Adoptive transfer of TCR and CAR-modified T-cells (left). CAR, chimeric antigen receptor; CC, cancer cell; CSC, cancer stem cell; TCR, T-cell receptor; TIL, tumor-infiltrating lymphocytes.

Genetic vaccination approaches (DNA/RNA/virus-based) induce somatic cell or DC expression of tumor antigens and their presentation in the context of MHC class I and II molecules. This can trigger a direct immune response against tumor cells (30). Initial clinical trials of RNA-based vaccine approaches are promising and suggest a superior side-effect profile over the other genetic vaccines (DNA/virus-based vaccines) (**Figure 2**, **Table 2**).

Over many years, the potent stimulatory effects of Toll-like receptors (TLRs) on the immune system have urged efforts aiming to develop immune vaccines that use TLR agonists as immunological adjuvants (31, 32). Motolimod (VTX-2337) and resiquimod (R848) are TLR-8 and TLR-7/TLR-8 agonists respectively, that deliver adjuvant-like signals to APCs. Both are derivatives of first generation immunomodulatory agents like imiquimod, which was originally approved by the US Food and Drug Administration (FDA) to treat genital warts and actinic keratosis. VTX-2337 and R848 are currently being investigated as potential immune system stimulators for the treatment of various tumor types (including CRC and mCRC). They might be particularly considered effective in combination therapies together with cancer cell lysate-based, dendritic cell-based, DNA molecules-based or peptide-based vaccines (31). The CD200 receptor (CD200R) inhibits immune activation upon binding to its ligand CD200 that is often expressed on tumor cells to diminish anti-cancer immune response (33, 34). Previous studies have shown that the intratumoral administration of R848 inhibits tumor growth and decreases CD200R expression on tumor-infiltrating immune cells in a syngeneic CT26 colon carcinoma mouse model. These results indicate that the antitumor activity of the TLR-7/TLR-8 agonist (R848) is mainly driven by an anti-CD200R effect causing changes in the tumor microenvironment (TME) (32).

DNA motifs containing unmethylated cytosine‐guanosine oligodeoxynucleotides (CpG‐ODN) have an immunostimulatory function and can induce antitumor immune responses mediated by the innate and the adaptive immune system through TLR‐9 signaling upon activation of APCs. CpG-ODNs promote the maturation of APCs and support the generation of antigen-specific B cells and cytotoxic T lymphocytes (35, 36). In humans, TLR‐9 is mainly expressed by B cells and plasmacytoid dendritic cells. Several experimental models have shown that immune modulation by a TLR‐9 agonist (e.g., CpG‐28 or MGN1703) can activate both innate and adaptive immunity resulting in a significant tumor rejection; particularly when injected directly into the tumor (37, 38). Additionally, intratumoral injection of CpG-ODN enhances the host’s response against cancer cells by reducing the immunosuppressive activity of myeloid-derived suppressor cells (MDSCs) (39). CpG-ODN treatment can also increase the TNF production in DCs or peripheral blood mononuclear cells (PBMCs) (40).

Multiple clinical studies have been initiated and first analyses suggest a certain efficacy of vaccination-based approaches in different tumor types. However, the overall clinical success has been low especially for CRC (41). To date, there are no clinically approved vaccination therapies in CRC or mCRC treatment, and those that are tested in different clinical trials can induce a therapeutic response only in a minority of patients (about 5–10%) (30).

The reasons why previous vaccination strategies did not produce satisfactory clinical response rates are mainly explainable by the decreased reactivity of the immune system to tumor-associated self-antigens due to tolerance and immunosuppressive mechanisms (30). In addition, previous research studies have used single Ag or combinations of only a few Ags as the basis for tumor vaccination strategies. However, according to most recent studies and guidelines multi-epitope vaccines are considered superior to single Ag based vaccines (41). There is also emerging evidence that tumor vaccination appears to be more effective when the tumor burden is still at a low level. Previous studies, however, were mostly performed on advanced cancer stages or metastatic forms (41).

## Passive Immunotherapy

### Antibodies

Monoclonal antibodies are the longest used form of immunotherapy. They are part of the treatment regimens for many tumors including mCRC. Antibodies, directed against the tumor or tumor-associated structures could mediate their effect *via* induction of cell death, activation of the complement system, activation of effector cells of the immune system *via* the Fc part of the antibody or opsonization (“labeling”) of tumor cells, and facilitation of phagocytosis by myeloid cells (42).

### Immune-Checkpoint Inhibitors

Checkpoint inhibitors (CHI) have revolutionized tumor therapies in recent years. Their discovery is considered as one of the most important immunotherapy innovations of the last decade. Several compounds targeting these molecules are already in development (43).

The immune system has numerous co-stimulatory and inhibitory signaling pathways that help to regulate the strength of an immune response and prevent autoimmune reactions. The inhibitory signaling pathways, so-called immune-checkpoints, cause downregulation of T-cell activation or effector function and play a central role to protect our body from excess immune and T-cell responses. Tumor cells use upregulation of immune-checkpoints to escape immune system recognition (immune evasion) and protect themselves from T-cells and the immune system. This knowledge led to the development of CHIs. These inhibitors are monoclonal antibodies (mAbs) directed against immune-checkpoint receptors or ligands; thereby, resolving the physiological “immune brakes.” Because CHI modulate the immune response, they clinically show a different type of response than conventional oncology therapeutics. CHI may initially lead to an apparent phase of tumor growth that is followed by tumor regression (44). The described side-effects of CHI can also largely be explained by the immune-stimulatory mode of action, which can cause a misguided immune response. However, CHI-side effects can be treated well (44).

Three representatives of checkpoint molecules play an essential role in oncology: PDCD1 [“programmed cell death protein 1 (PD-1)”], CD247 [“programmed cell death-ligand 1 (PD-L1)”], and CTLA4 (“cytotoxic T-lymphocyte antigen-4”). All three molecules were tested and validated as targets for blocking antibodies. CTLA4 belongs to the first generation of CHI. CTLA4 is expressed on activated cytotoxic T-cells and acts as the antagonist of the costimulatory receptor CD28 that is required in T-cell activation. The blockade of CTLA4 thus leads to a *de novo* generation and expansion of T-cells. The mAb, ipilimumab, binds to CTLA4 and subsequently activates antitumoral effects in the early phase of T-cell activation within the lymph nodes. Another CTLA4 inhibitor, tremelimumab, is currently in clinical development (45).

The target genes of the second generation of CHI are the checkpoint receptor PDCD1 and its ligands CD247 (B7-H1) and PDCD1LG2 (B7-DC or PD-L2). PDCD1 plays a key role in the regulation and maintenance of the balance between T-cell activation and immune tolerance (45). The PDCD1/CD247 axis plays a pivotal role in the effector function of T-cells, i.e., in T-cells residing within (tumor) tissue. It protects cells from excessive T-cell activation by the expression of CD247 on the cell surface that interacts with PDCD1 expressed by T-cells. Thus, the blockade of the PDCD1/CD247 axis would reactivate an existing T-cell response. Several mAbs against PDCD1 (pembrolizumab, lambrolizumab, nivolumab, and pidilizumab) and its major ligand CD247 (BMS-936559, MPDL3280A, etc.) are currently in clinical development to target a variety of tumors including mCRC. The most advanced substances are pembrolizumab and nivolumab. Numerous other checkpoint molecules are the subject of intensive preclinical research: Hepatitis A virus cellular receptor 2 [HAVCR2; known as T-cell membrane protein 3 (TIM-3)], lymphocyte activation gene 3 (LAG3) (**Table 2**).

Little oxygen is beneficial for tumors by preventing tumor cells from T-cell interaction. Moreover, hypoxia counteracts the desired effects of CHI, such as PDCD1 or CTLA4 inhibitors leading to CHI resistance of tumor cells (46). In this regard, the substance evofosfamide (TH-302), an alkylating prodrug that is activated by a lack of oxygen supply, is currently being clinically tested (47).

It is already evident that some types of tumors such as melanoma, lung, kidney, or bladder carcinoma and Hodgkin’s lymphoma respond better to immunotherapy with CHI than other types of tumors (e.g., tumors of the gastrointestinal tract and pancreatic carcinoma) (30). The two PDCD1 inhibitors (pembrolizumab and nivolumab) have been evaluated alone or in combination with a CTLA4 inhibitor in patients with chemorefractory mCRC in the frame of several clinical studies (**Table 2**). As results, patients had an improved therapy response rate and around 60–70% disease control (48, 49). Interestingly, the response to CHI was irrespective of CD247 expression within tumor cells. Further, the response rate was independent from the history of Lynch syndrome and *BRAF* or *KRAS* mutation status (50). Although CHI (including PDCD1 blockade or anti-CTLA4) in patients with dMMR/MSI-H mCRC significantly increase the antitumor activity of tumor specific CD8^+^ T-cells with highly durable tumor response, they are associated with virtually no activity in patients with pMMR/non-MSI-H mCRC (51, 52).

Tumor-infiltrating CD4^+^Foxp3^+^ regulatory T (Treg) cells are known as potent immunosuppressive cells. Treg cells represent one of multiple TME components that help cancer cells to evade the immune system (53, 54). Accumulation of Tregs within tumor tissues and the subsequent high ratio of Tregs to effector T (Teff) cells, is correlated with poor prognosis of cancer patients suffering from different types of malignancies, including CRC (55). Thus, several cancer immunotherapy approaches purging the activity of CD4^+^Foxp3^+^ Treg cells by either depletion of or down-regulating their immunosuppressive function using immune-checkpoint inhibitors such as anti-CTLA-4 monoclonal antibody therapy. This approach has become an effective cancer immunotherapy attributing to depletion of Tregs in tumors (54).

## Combination Therapy

Despite good clinical results, many patients do not respond to single CHI treatment. However, it might be possible to optimize overall survival rates through appropriate therapy combinations. The spectrum of combination partners ranges from further CHI and vaccines, *via* radiotherapy and chemotherapies, to targeted therapeutic approaches (56). The combination of ipilimumab (CTLA4 inhibitor) and nivolumab (PDCD1 inhibitor) results in a synergistic effect improving progression-free survival compared to monotherapy with nivolumab or ipilimumab in tumors with mismatch-repair deficiency (CRC and mCRC) (48, 51). A cohort of 119 patients showed disease control rates of around 80% and overall response rates of more than 50% upon combination treatment using both CHI. Overall survival rates improved from 60 to 85% compared to monotherapy.

A comparable combination study with pembrolizumab has already been initiated (**Table 2**). In addition, combination treatment trials with other modulators of inhibitory (e.g., LAG3, HAVCR2, BTLA, and “B and T lymphocyte attenuator”) and stimulatory molecules [e.g., ICOS, “inducible T-cell costimulator”; TNFRSF9 (4-1BB)] are under investigation (56).

## Immune-Checkpoints as Prognostic Biomarkers

Selection markers for targeted therapy with CHI are currently being intensively researched. However, no clear immunological or tumor-specific characteristics could be identified that clearly predict responsiveness to CHI in CRC and mCRC. According to current knowledge, the therapy with CHI of both the CTLA4 and the PDCD1/CD247 signaling pathway is particularly well responsive to tumors that have a high mutational load (57, 58). Tumors that carry genetic defects in their DNA repair machinery (“MMR defect”) and consequently present high mutation rates are much more responsive to anti-PDCD1 therapy than tumors without MMR defects (57). For the PDCD1/CD247 system, most biomarker studies are concerned with the CD247 expression pattern. Looking at all studies across all tumor entities, patients whose tumors express CD247 appear to respond better to PDCD1 blockade than patients without CD247 expression. Nevertheless, CD247 negative patients also respond to CD247 checkpoint blockade. Therefore, according to current knowledge, CD247 cannot generally be recommended as a selection marker for PDCD1 blockade (56). However, recent data suggest that a patient’s CD247 status may play a role in deciding whether to use dual checkpoint inhibition (48). Determining the immunogenicity of the tumor environment could also be important for the choice of tumor therapy. For example, non-immunogenic tumors (“cold tumors”) are more likely to benefit from combination therapies (56).

Since CHI activate the adaptive immune system, a tumor-specific immune response is possible, which may be independent of the histological subtype and the type and number of prior therapies. Currently, T-cell therapies alongside CHI are regarded as great hope carriers of immuno-oncology, even though they are still partially in the developmental phase (45).

## T-Cell Therapies

T-cell based immunotherapies are referred to as “live drugs”: cell preparations that contain T-cells are currently being clinically researched by oncologists. Adoptive cell transfer (ACT) with T-cells is a highly personalized form of therapy in which patients are endowed with specific T-cells that have direct antitumoral activity. In contrast to vaccination or CHI, the immune system equipped with effector T-cells can exert its antitumoral function immediately. Currently, three classes of effector T-cells are in the process of being approved: tumor-infiltrating lymphocytes (TIL), genetically modified T-cells with a chimeric antigen receptor (CAR), and T-cells targeted with a specific genetically modified T-cell receptor (TCR). In ACT approaches, T-cell lymphocytes are isolated from tumors of individual patients, modified, selected and expanded *ex vivo*, then reinfused into the patient (**Figure 2**).

### Tumor-Infiltrating Lymphocytes

It has been shown that the presence of TILs is associated with a good prognosis of cancer and that TILs isolated from tumor tissue show a selective antitumor activity (59).

Preclinical and clinical studies on TIL’s ACT show clinical response rates and sustained remission rates in metastatic CRC (60). Recently, modern molecular analysis has shown that melanoma regression-inducing TILs are polyclonal T-cell populations that recognize different neoantigens on tumor cells (61). This finding confirms the long postulated assumption that neoantigens on tumor cells are the main target of immune system recognition (62) and explains the good response rates toward ACT therapies with TILs in melanoma, a tumor entity with one of the highest mutational burdens, or in MSI-H patients (6). Therefore, the establishment of methods for the selection of neoantigen reactive TILs may lead to improved therapeutic success also in other tumor types.

### Genetically Modified T-Cells

The idea of ​​genetically modified T-cells was developed to target directly and more specifically tumor cells with activated T-cells that have specific T-cell receptors (TCRs). The production of TCR-modified T-cells is carried out by transfection or transduction of autologous T-cells with vectors, which code for tumor-specific α/β TCRs. They can be isolated by different methods (63, 64). Any patient whose tumor expresses the tumor antigen and the correct MHC allele may benefit from such therapy approaches. In the complementary strategy of CAR-modified T-cells, CAR genes are expressed in autologous T lymphocytes. CAR are transmembrane single-chain fusion proteins and the centerpiece of which is an extracellular antibody binding site, which, in contrast to conventional TCR, recognizes an intact surface structure on tumor cells. This Ab binding site is linked to one to three TCR intracytoplasmic signaling regions *via* a transmembrane region. They serve to initialize T-cell activation signals. CAR modified T-cells are activated and proliferate MHC-independent *in vivo* after exposure to the antigens. This can lead to tumor cell lysis and the formation of an Ag-specific immune memory (64). Today it is assumed that especially T-cells in early stages of differentiation (naive and central memory T-cells) are particularly suitable for ACT with gene-modified T-cells (65). The cancer researchers have succeeded in using genetic engineering to produce third-generation CAR receptors, which can transmit at least three signals, or the fourth generation, known as TRUCK, which can be combined with cytokines (66, 67).

To date, CAR therapy has been less successful in solid tumors. In order to be able to better control genetically modified T-cells, it would be advantageous to be able to switch them on and off after infusion. Therefore, Anja Feldmann, Dresden-Rossendorf, and their colleagues have developed special CAR-T-cells that are initially inactive and can be temporarily “armed” only when needed by an externally added factor against tumors. These are short-lived mono- or bispecific molecules, for example against the growth factor EGFR (“epidermal growth factor receptor”), which make the connection between the cytotoxic T-cells and the tumor cells and thus the T lymphocytes to the cancer cells. Such UniCAR T-cells can be inactivated again by omitting the activating factors (68).

Several pilot studies with TCR-modified T-cells indicate a good response of various solid and hematological tumors to this therapeutic strategy; these include melanomas, synovial sarcomas, multiple myelomas, colorectal and hepatocellular carcinomas (64). Clinical studies with CAR-modified T-cells have so far been conducted predominantly with anti-ERBB2 specificity for the treatment of CRC/mCRC (69). However, ACT with gene-modified T-cells has often been associated with side effects. Thus, strong immune responses against healthy target tissue could be elicited if the target antigen is not expressed exclusively on tumor cells (“on-target toxicity”) or result in cross-reactivities. Neurotoxicities are other frequently observed side effects (60, 64). The severe side effects of treatment-induced massive T-cell activation and associated excessive cytokine secretion, which occur in particular during CAR-modified T-cell therapies, can now be treated well with tocilizumab, a mAb directed against the interleukin-6 receptor (IL6R).

In solid tumors, CAR modified T-cell therapies have not been widely accepted. This is due to the lack of antigens expressed exclusively on tumor cells. In addition, oncological T-cell therapies are extremely costly. Initial clinical trials to combine ACT with other immuno-oncological therapeutics (CHI and vaccines) or targeted therapies are passing different clinical trials.

## Immunotherapies Targeting Cancer Stem Cells

CSCs are resistant to conventional chemotherapies due to their quiescent cell states and are considered the main drivers of disease relapse and cancer metastasis (70, 71). In recent studies, a diversity of new antigens has been described that are expressed on colorectal CSCs but are absent in the tumor bulk (non-CSC population) and healthy tissues. These antigens provide promising targets to eradicate CSCs by directed T-cell responses thereby disrupting the generation of new cancer cells. In this regard, the antigen ASB4 has recently been described to be upregulated in colorectal CSCs. Interestingly, treatment with adoptively transferred effector CD8^+^ T-cells specifically targeting ASB4 led to the elimination of CSCs and suppressed tumor growth *in vivo* (71). Glycoprotein A33 (GPA33) was found to be universally expressed on CSCs and non-CSCs populations using a panel of cancer stem-like cell lines derived from human CRC specimens. Treatment with a bispecific GPA33–CD3 monoclonal antibody (MGD007), recruiting human T-cells, induced lysis of GPA33 expressing CRC cells and reduced tumor growth *in vivo* in NOD/SCID mice subcutaneously injected with a 1:1 mixture of colorectal cancer cell lines (LS174T and Colo205) with purified T-cells (72). The surface markers CD133 and CD44 have been shown to enable the discrimination of colorectal CSCs and non-CSCs (73, 74). Radiotheranostic targeting of colorectal CSCs using Prominin 1 (PROM1; known as CD133) and CD44 monoclonal antibodies labeled with radioodine led to a significant inhibition of tumor growth and prolonged mean survival of xenografted mice injected with HT29 CRC cell line (75). Besides immunotherapy-based approaches to target CSCs in CRC/mCRC, novel options are emerging that might play a pivotal role in future treatment regimens targeting and eliminating CSCs. Tankyrase-inhibitors have been shown to effectively reduce the CD44-positive COLO-320DM cell population resembling CSC properties. Moreover, co-treatment of tankyrase-inhibitors with Irinotecan significantly decreased tumor growth of COLO-320 xenograft tumors in immunodeficient mice and showed higher efficiency than single treatment (76). Mithramycin-A (Mit-A) treatment, an antibiotic that inhibits the binding of transcription factors to DNA, led to a reduction in size and numbers of tumor spheroids derived from the CRC cell lines, HT29, HCT116, and KM12 compared to standard treatment with 5-fluorouracil and oxaliplatin (FUOX). In addition, PROM1 expression and ALDH activity of tumor spheroids were downregulated upon Mit-A treatment demonstrating a direct suppressive effect on cancer cell stemness (77, 78).

## Gut Microbiome and Immunotherapies

**Figure 3:**
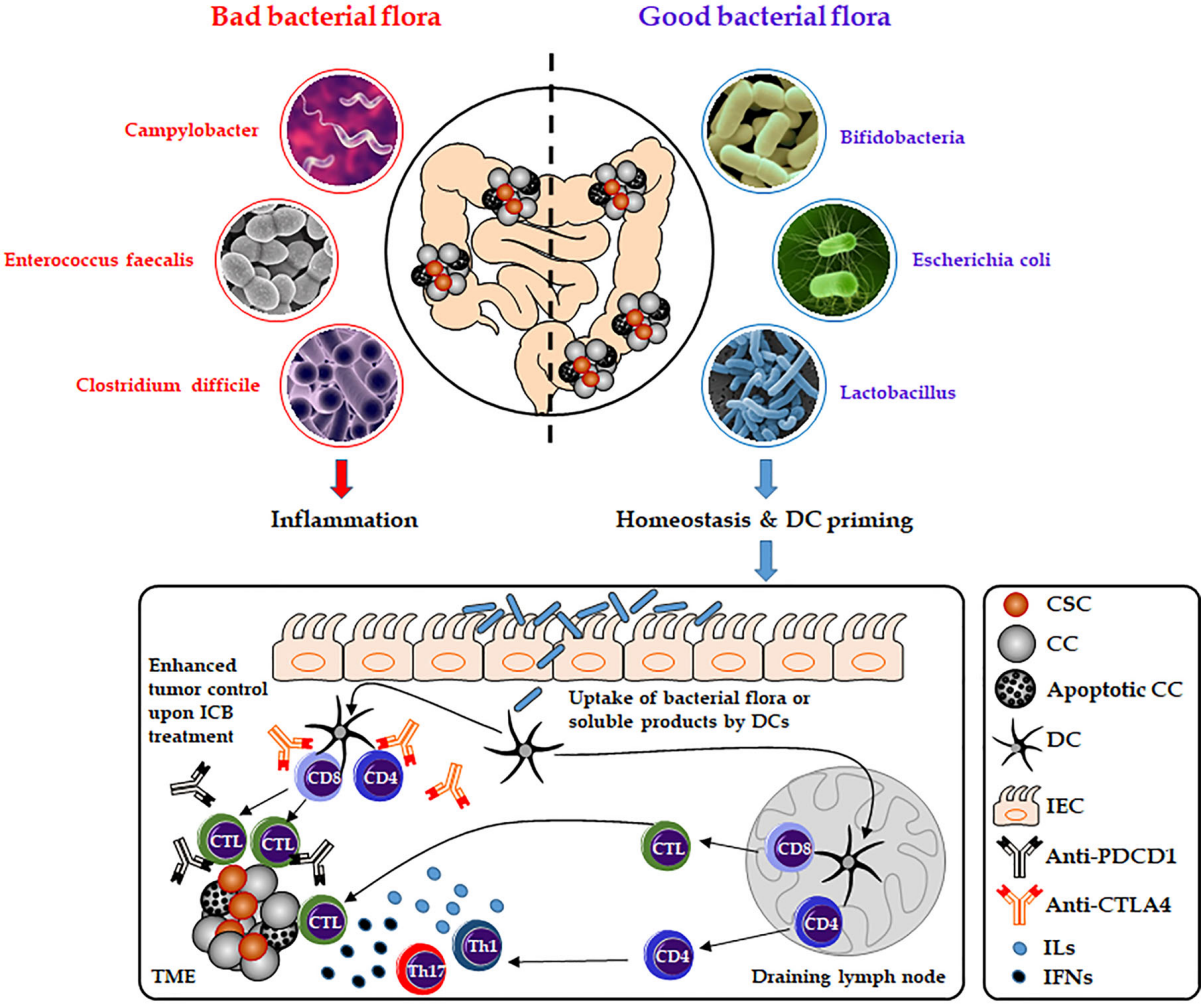
Role of the gut microbiome on immune-checkpoint blockade therapies. Composition of the gut microbiota can affect host antitumor immunity. Uptake of distinct good bacterial flora (e.g., Bifidobacteria, *Escherichia* *coli*, and *Lactobacillus*) or bacteria-derived products by DCs can enhance the antigen-processing steps and presentation by DCs and thereby affect the response to CHI therapies (e.g., anti-PDCD1 or anti-CD247). As a result, this will lead to the activation of cytotoxic T lymphocytes or more increased secretion of interleukins and interferons (e.g., IL17 or INFG) by activated T helper cells. However, bad bacterial species (e.g., *Campylobacter*, *Enterococcus* *faecalis*, and *Clostridium* *difficile*) might have a negative effect on DC activation and CHI therapies by inducing inflammation and disrupting the gut microbiome homeostasis. CC, cancer cell; CSC, cancer stem cell; CTL, cytotoxic T lymphocyte; DC, dendritic cell; ICB, immune-checkpoint blockade; IEC, intestinal epithelial cell; IFNs, interferons; ILs, interleukins; Th, T-helper cell; TME, the tumor microenvironment.

There is emerging evidence that the gut microbiome plays a pivotal role in carcinogenesis, immunity and might affect cancer response to immunotherapies (79, 80). In CRC, the interactions between gut commensals, immune cells, and cancer cells build a complex and not fully understood network that might drive or inhibit cancer progression depending on various factors such as the composition of the patient’s microbiome (**Figure 3**). In 2009, Wu et al. for the first time described that colonization with entertoxigenic *Bacteroides fragilis* (BTF) and BTF toxin-mediated colitis followed by recruitment of T helper type 17 (Th17) cells increase the chances of inflammation-induced colorectal cancer (81). The state of the gut microbiome has also been linked to CHI response in melanoma and renal cell carcinoma patients. In this regard, Chaput et al. described better response rates of patients suffering from metastatic melanoma to treatments with the CTLA4 inhibitor, ipilimumab, based on an intact and stable state of the gut microbiome. Patients whose microbiota was enriched by *Faecalibacteria* or other Firmicutes survived significantly longer and showed higher rates of ipilimumab-mediated induction of T-cells compared to patients without evidence of gut colonization by *Faecalibacteria* (82). Moreover, preclinical and clinical studies revealed that treatment with antibiotics disrupting the equilibrium of the normal gut microbiome result in a compromised efficacy of anti-PDCD1 therapies. RET melanoma and MCA-205 sarcoma mice that were pre-treated with antibiotics survived significantly shorter undergoing anti-PDCD1 and anti-CTLA4 treatment compared to mice without antibiotic pre-treatment. Moreover, in an anti-PDCD1/CD247 treated cohort of 140 non-small cell lung cancer and 67 renal cell carcinoma patients, reduced overall survival could be observed in those that underwent additional antibiotic therapies (83).

Besides the microbiome, many other exogenous and endogenous factors affect cancer progression and therapy response. However, the interplays between these exposures and their effects on cancer progression and therapy response are not well investigated and are therefore an emerging field of scientific interest. The transdisciplinary discipline of molecular pathological epidemiology (MPE) uses molecular pathological signatures to elucidate these complex interactions on disease progression and provide new concepts of disease prediction and treatment. In CRC research, MPE projects led to significant progress in the understanding of cancer heterogeneity between different CRC subtypes based on the analysis and wholesome evaluation of genetic, epigenetic and microbial statuses of CRC patients and have a great potential to improve precision based medicine in the future (84–88).

## Conclusion

Within a few years, immunotherapy has become a successful oncological therapeutic strategy. It has the potential to induce sustained tumor remission in various tumor entities including CRC/mCRC, which could significantly improve the overall survival of cancer patients.

Tumor vaccination is highly complex and the optimal combination of antigens, adjuvants and administration routes is not yet clearly identified. From today’s perspective, the future of oncological vaccination strategies lies in the development of targeted oncological vaccines based on patient-specific neoantigens and in the combination of various therapeutic strategies such as CHLs, CAR T-cells, or adaptive cell therapies.

Among other developed immunotherapy strategies, checkpoint inhibitors showed a great success rate as a potential immuno-oncological therapy, in particular for dMMR mCRC. However, to date, the economic impact of these therapies largely remains unknown. It is shown that although both single or combination CHI were superior to chemotherapy in dMMR mCRC, they were less cost-effective.

In T-cell therapies, the response rates vary greatly depending on the underlying disease. Whether and to what extent this benefit can be transferred to other tumors will have to be shown in further studies. In the near future, modern molecular biology techniques might enable the development of patient-specific neoantigen-specific receptors for ACT.

While the role of tumor vaccination is currently unclear, the clinical successes of CHI and ACT with T-cells show that therapeutic manipulation of the immune system represents a new successful oncological treatment strategy for CRC. Co-targeting of CSCs as the disease initiating and maintaining population of cancer cells might increase the success rate of current CRC treatment approaches. Immuno-oncology has the potential to induce sustained tumor regression and significantly improve overall survival in many tumors including mCRC.

## Author Contributions

SF and RR wrote the manuscript. All authors contributed to the article and approved the submitted version.

## Conflict of Interest

The authors declare that the research was conducted in the absence of any commercial or financial relationships that could be construed as a potential conflict of interest.
